# Intermittent methionine restriction reduces IGF‐1 levels and produces similar healthspan benefits to continuous methionine restriction

**DOI:** 10.1111/acel.13629

**Published:** 2022-05-15

**Authors:** Jason D. Plummer, Jay E. Johnson

**Affiliations:** ^1^ Department of Biology Orentreich Foundation for the Advancement of Science Cold Spring New York USA

**Keywords:** aging, intermittent, longevity, lifespan, metabolism, obesity, IGF‐1, mice

## Abstract

A sustained state of methionine restriction (MR) dramatically extends the healthspan of several model organisms. For example, continuously methionine‐restricted rodents have less age‐related pathology and are up to 45% longer‐lived than controls. Promisingly, MR is feasible for humans, and studies have suggested that methionine‐restricted individuals may receive similar benefits to rodents. However, long‐term adherence to a methionine‐restricted diet is likely to be challenging for many individuals. Prompted by this, and the fact that intermittent variants of other healthspan‐extending interventions (*i*.*e*., intermittent fasting and the cyclic ketogenic diet) are just as effective, if not more, than their continuous counterparts, we hypothesized that an intermittent form of MR might produce similar healthspan benefits to continuous MR. Accordingly, we developed two increasingly stringent forms of intermittent MR (IMR) and assessed whether mice maintained on these diets demonstrate the beneficial metabolic changes typically observed for continuous MR. To the best of our knowledge, we show for the first time that IMR produces similar beneficial metabolic effects to continuous MR, including improved glucose homeostasis and protection against diet‐induced obesity and hepatosteatosis. In addition, like continuous MR, IMR confers beneficial changes in the plasma levels of the hormones IGF‐1, FGF‐21, leptin, and adiponectin. Together, our findings demonstrate that the more practicable intermittent form of MR produces similar healthspan benefits to continuous MR, and thus may represent a more appealing alternative to the classical intervention.

## Graphical abstract

Continuous methionine restriction (MR) extends the healthspan of a number of model organisms, including rodents. Here, we show that 3 days of stringent MR per week (aka, intermittent MR; IMR) are sufficient to protect mice against diet‐induced obesity, hepatosteatosis, and glucose intolerance, as well as confer additional metabolic health benefits. As a result, IMR may be a preferable alternative to continuous MR.

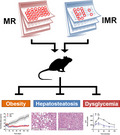

**Abbreviations**
CF: control diet
CR: calorie restriction
ELISA: enzyme‐linked immunosorbent assay
GH: growth hormone
IMR: intermittent methionine restriction
KD: ketogenic diet
MET: methionine
MR: methionine restriction

## 1 INTRODUCTION

Continuous methionine restriction (MR) is known to improve mammalian healthspan. Rats fed a methionine‐restricted diet have a reduced incidence of age‐related pathologies and are up to 45% longer‐lived than control‐fed littermates; methionine‐restricted mice receive similar benefits (Miller et al., 2005; Orentreich et al., 1993; Richie et al., 1994). Beyond improving overall survival, MR confers several metabolic health benefits to rodents, including reduced white adipose tissue accumulation, protection against hepatosteatosis, improved glycemic control, and altered plasma levels of multiple nutrient‐sensing hormones that regulate metabolism and healthspan (Malloy et al., 2006, 2013; Miller et al., 2005; Yang et al., 2019). Indeed, such metabolic benefits are so robust that continuous MR completely protects rodents against the diet‐induced obesity that results from consumption of a high‐fat diet meant to approximate the human Western diet (Ables et al., 2012).

Previous studies from our laboratory and others have suggested that the response to MR is conserved and that continuous MR is likely to confer healthspan benefits to humans similar to those observed for rodents (Dong et al., 2018; Gao et al., 2019; Johnson & Johnson, 2014; Olsen et al., 2020). Given that the vegan diet is low in both total protein and free amino acids, continuous MR is possible for humans. Unfortunately, long‐term compliance to such a diet is likely to be challenging. Consequently, we and others in the aging field have sought to develop simpler and/or more practicable interventions that produce similar healthspan benefits to MR.

Multiple studies have demonstrated that intermittent variants of well‐characterized dietary interventions can have healthspan‐extending effects comparable to, and in some cases, greater than, those of their continuous counterparts. Continuous calorie restriction (CR), which is characterized by a 15%–60% reduction in caloric intake, is associated with extended healthspan and improved body condition in a number of model organisms (Weindruch & Sohal, 1997). Intermittent CR, defined as 24 h of *ad libitum* food consumption followed by 24 h of CR, is just as effective as continuous CR at reducing adiposity and improving body condition in mammals (Varady, 2011; Varady & Hellerstein, 2007). In addition, as compared with continuous CR, more lean body mass is preserved by intermittent CR, indicating that the latter intervention might not only be easier to sustain, but also preferable in terms of physiological outcomes. Intermittent fasting, an intervention that is similar to intermittent CR, but marked by a total, rather than partial, reduction in calories during the restriction period, produces similar benefits (Varady, 2011). Another extensively studied healthspan‐promoting dietary intervention is the ketogenic diet (KD), which is high in fat, low in carbohydrates, and features adequate total protein and free amino acids (Newman & Verdin, 2014). The composition of this diet forces the body to rely on the metabolism of fat, rather than carbohydrates, and the ketone bodies produced are associated with positive health outcomes (Roberts et al., 2017). Interestingly, a recent study found that an intermittent variant of this diet (*i*.*e*., cyclic KD) is actually more effective than continuous KD at conferring some of the benefits associated with this intervention (Newman et al., 2017). Together, these observations prompted us to ascertain whether an intermittent variant of MR might confer to mice the healthspan benefits typically associated with continuous MR.

To test whether intermittent MR (IMR) is capable of producing similar healthspan benefits to continuous MR, we developed two increasingly stringent dietary interventions (IMR1 and IMR2), characterized by alternating periods of methionine repletion (4 days) and methionine restriction (3 days). We assessed the relative abilities of these interventions to protect against the diet‐induced obesity typically observed for animals fed a high‐fat, methionine‐replete diet. We also measured several other physiological parameters known to be positively altered by continuous MR. Here, we show that only 3 days of MR per week is sufficient to produce the healthspan benefits typically associated with continuous MR, including dramatically lower white adipose tissue accumulation, protection against hepatosteatosis, improved glycemic control, elevated ketone body production, and altered plasma levels of IGF‐1, FGF‐21, leptin, and adiponectin. Together, our results support IMR as a more practicable form of MR that confers similar health benefits to the continuous intervention.

## 2 RESULTS

### 2.1 Like continuous MR, IMR protects male mice against diet‐induced obesity

**FIGURE 1:**
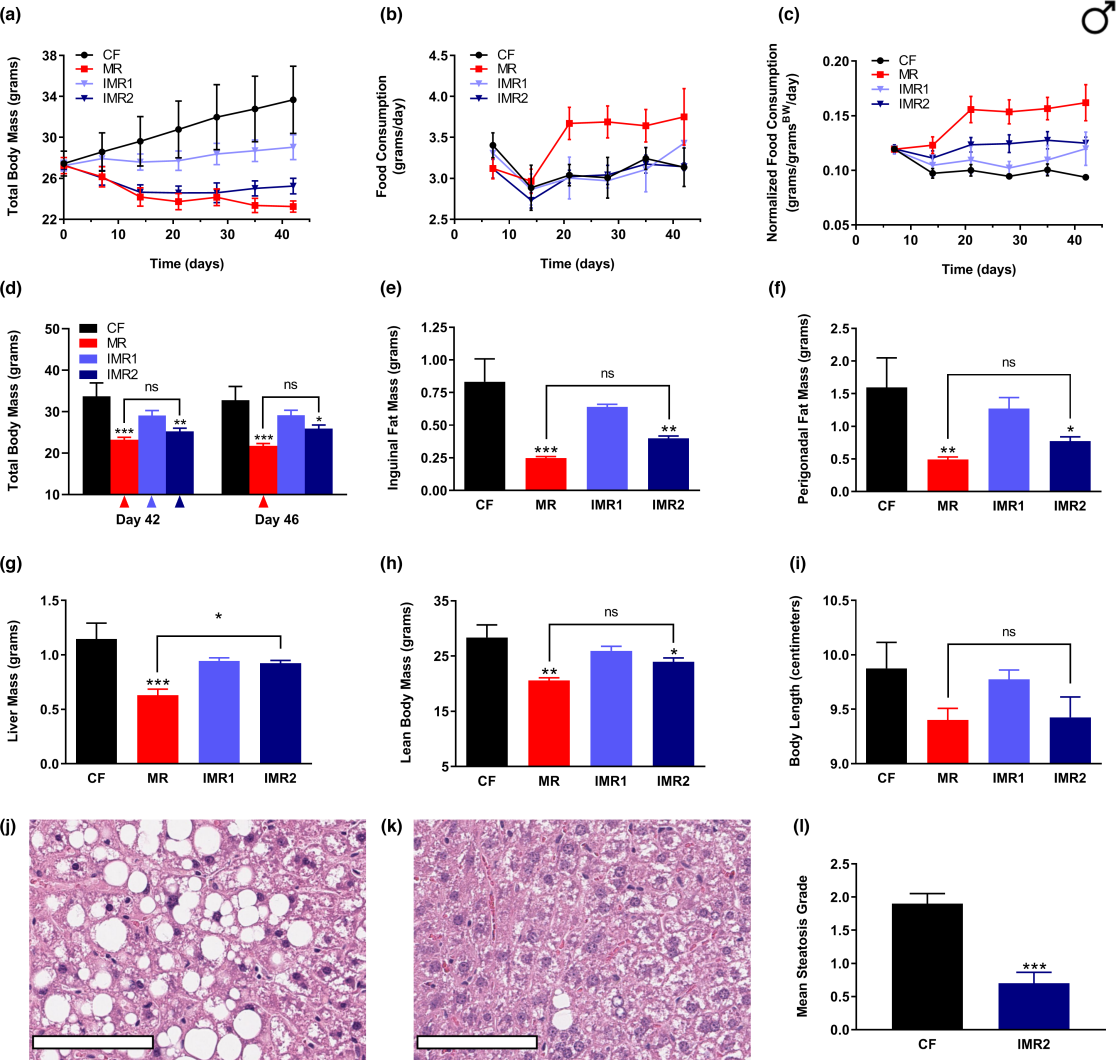
IMR of male mice produces similar healthspan benefits to continuous MR and prevents liver steatosis. Comparisons over time of average values for (a) total body mass, (b) food consumption, and (c) food consumption normalized to total body mass for control‐fed (CF; black circles) or continuously methionine‐restricted (MR; red squares) male mice, as well as animals subjected to IMR (IMR1; light blue triangles) or a more stringent IMR regimen (IMR2; dark blue triangles). For simplicity, food consumption graphs (b‐c) show values integrated over 7 days of feeding; expanded graphs are presented in Figure S2. Average values at conclusion of the experiment (~6 weeks) are also shown for (d) total body mass, (e) mass of inguinal fat pads, (f) mass of perigonadal fat pads, (g) liver mass, (h) lean body mass, and (i) body length. Representative photomicrographs (200× magnification) of the livers of mice fed either (j) the control diet or (k) the IMR2 regimen for ~16 weeks, as well as (l) a comparison of the mean steatosis grades of these animals. For panel D, the colored triangle denotes that weighing of the indicated group occurred following a period of MR. For panels a‐i and l, bars denote SEM. For panels j‐k, bars represent 100 µm. For panels d‐i and l, statistically significant differences (as compared with CF values) are indicated (*p < 0.05; **p < 0.01; ***p < 0.001). For panels d‐i, statistically significant differences between MR and IMR2 values are either indicated (*p < 0.05) or absent (ns). For panels a‐i, *N* = 4 for all groups; for panel l, *N* = 6 for both groups

In order to assess the ability of IMR to confer the benefits typically observed for continuous MR, we made use of the diet‐induced obesity model. Specifically, this model tests the ability of an intervention to protect mice against the increased adiposity, impaired glycemic control, and other poor health outcomes that result from chronic feeding with high‐fat diets; such diets provide nearly 60% of total calories from fat. In this context, we developed two intermittent variants of MR characterized by decreasing methionine content (Figure S1). The first of these (IMR1) features a cycle of 4 days of *ad lib* feeding of a methionine‐replete control diet (0.86% methionine), followed by 3 days of *ad lib* feeding of an isocaloric methionine‐restricted diet (0.12% methionine). The second IMR regimen (IMR2) also features a cycle of 4 days of *ad lib* feeding of the control diet, but in this case, is followed by 3 days of *ad lib* feeding of an isocaloric diet that is totally devoid of methionine. The time‐integrated methionine concentrations that result from these feeding conditions are 0.54% (IMR1) and 0.49% (IMR2), respectively. None of the diets utilized contain cysteine, as is typical for chemically‐defined rodent diets. For the initial study, wild‐type male C57BL/6J mice were fed, as follows, for a period of 6.5 weeks: 1) continuous control diet (CF), 2) continuous methionine‐restricted diet (MR), 3) intermittent regimen IMR1, and 4) intermittent regimen IMR2. Body mass and food consumption measurements were made regularly until the end of the experiment (Figure 1a‐c, Figure S2), at which point animals were sacrificed and multiple parameters representative of overall body condition and metabolism were assessed (Figure 1d‐i). Inguinal and perigonadal fat pads were surgically resected and weighed (Figure 1e‐f), as were livers (Figure 1g), which are prone to lipid accumulation (*i*.*e*., steatosis) in animals fed a high‐fat diet. Using this approach, we found that the more stringent IMR2 regimen was similarly effective to continuous MR at protecting male mice against the dramatic weight gain observed for control‐fed animals (Figure 1a,d). Further, animals subjected to IMR2 showed significantly less inguinal adiposity than controls, similar to continuously methionine‐restricted littermates (MR, 70%; IMR, 52%; Figure 1e). Interestingly, identical trends were observed for the accumulation of perigonadal adipose tissue (MR, 70%; IMR, 52%; Figure 1f). Even when normalized to lean body mass, the same inhibitory effect of the IMR2 regimen on adiposity was observed (Figure S3a‐b). With respect to total body mass measurements, as well as measurements of adiposity, animals subjected to IMR1 showed phenotypes intermediate to those of both control‐fed animals and littermates undergoing IMR2 (Figure 1a,d‐f), although no statistically significant differences (as compared with controls) were observed. However, the robust effects of the more stringent IMR2 regimen on male mice demonstrate the efficacy of IMR. At the end of the experiment (Day 46), the average total body mass of animals subjected to IMR2 was broadly similar to that of continuously methionine‐restricted animals (25.9 g vs 21.8 g; Figure 1a,d), and much less than that of controls (32.8 g). Notably, the observed ~4 g difference in average body mass was due almost entirely to the greater lean body mass of animals subjected to IMR2 as compared with continuously methionine‐restricted littermates (24.0 g vs 20.6 g; Figure 1h). This finding suggests that male mice undergoing IMR might not experience the same degree of growth inhibition as what is typically caused by continuous MR (Ables et al., 2012; Miller et al., 2005; Orentreich et al., 1993), although the observed difference in the average lean body mass of intermittently methionine‐restricted males and their continuously methionine‐restricted counterparts is not quite statistically significant (Figure 1h). With respect to the measurements of overall body length (Figure 1i), we failed to detect any significant effects of either intermittent or continuous MR. An interesting difference between intermittently and continuously methionine‐restricted male mice was that only the latter possessed small livers, an observation that was true whether or not liver mass values were normalized to lean body mass (Figure 1g, Figure S3c). To confirm that the observed differences in adiposity and body condition in methionine‐restricted animals were due to these interventions rather than a putative calorie reduction in the event that animals found the diets unpalatable, we assessed the rate of food consumption for all four regimens. The IMR regimens were consumed at a rate essentially equivalent to the control diet (Figure 1b, Figure S2a‐b). Consistent with previous findings, animals subjected to continuous MR consumed their food at a greater rate. When normalized to body size, both IMR regimens resulted in rates of food consumption intermediate to those of control‐fed and continuously methionine‐restricted animals (Figure 1c, Figure S2c‐d). Thus, male mice undergoing IMR were not calorie‐restricted, confirming that the protection against diet‐induced obesity observed was entirely due to periodic restriction of methionine.

### 2.2 IMR prevents liver steatosis

Given that animals maintained on a high‐fat diet develop liver steatosis (marked by an accumulation of triglyceride‐containing lipid droplets in hepatocytes) and that continuous MR guards against this condition (Malloy et al., 2013; Yang et al., 2019), we sought to determine whether IMR might confer similar protection. Toward this end, we performed liver histological analyses to determine the relative extent of steatosis in the livers of male mice undergoing the IMR2 regimen for 16.5 weeks. As expected, the livers of control‐fed mice showed a preponderance of hepatocytes containing lipid droplets (Figure 1j), whereas the livers of littermates subjected to IMR showed a normal architecture relatively free of such accumulations (Figure 1k). Grading the extent of steatosis (as a function of the proportion of fatty hepatocytes occupying the parenchyma) revealed that the IMR2 regimen conferred a 63% lower mean steatosis grade as compared with controls (Figure 1l). While it is known that continuous MR both protects against liver steatosis and results in smaller sized livers (Ables et al., 2012; Malloy et al., 2013; Yang et al., 2019), we recently observed that another healthspan‐promoting dietary intervention (*i*.*e*., selenium supplementation) operates similarly in that it has a robust anti‐adiposity effect and produces smaller livers as compared with controls (Plummer et al., 2021). However, IMR only has the former activity, as liver mass was not significantly affected by this regimen (Figure 1g, Figure S3c).

### 2.3 Like continuous MR, IMR confers beneficial metabolic and plasma hormone changes to male mice

**FIGURE 2:**
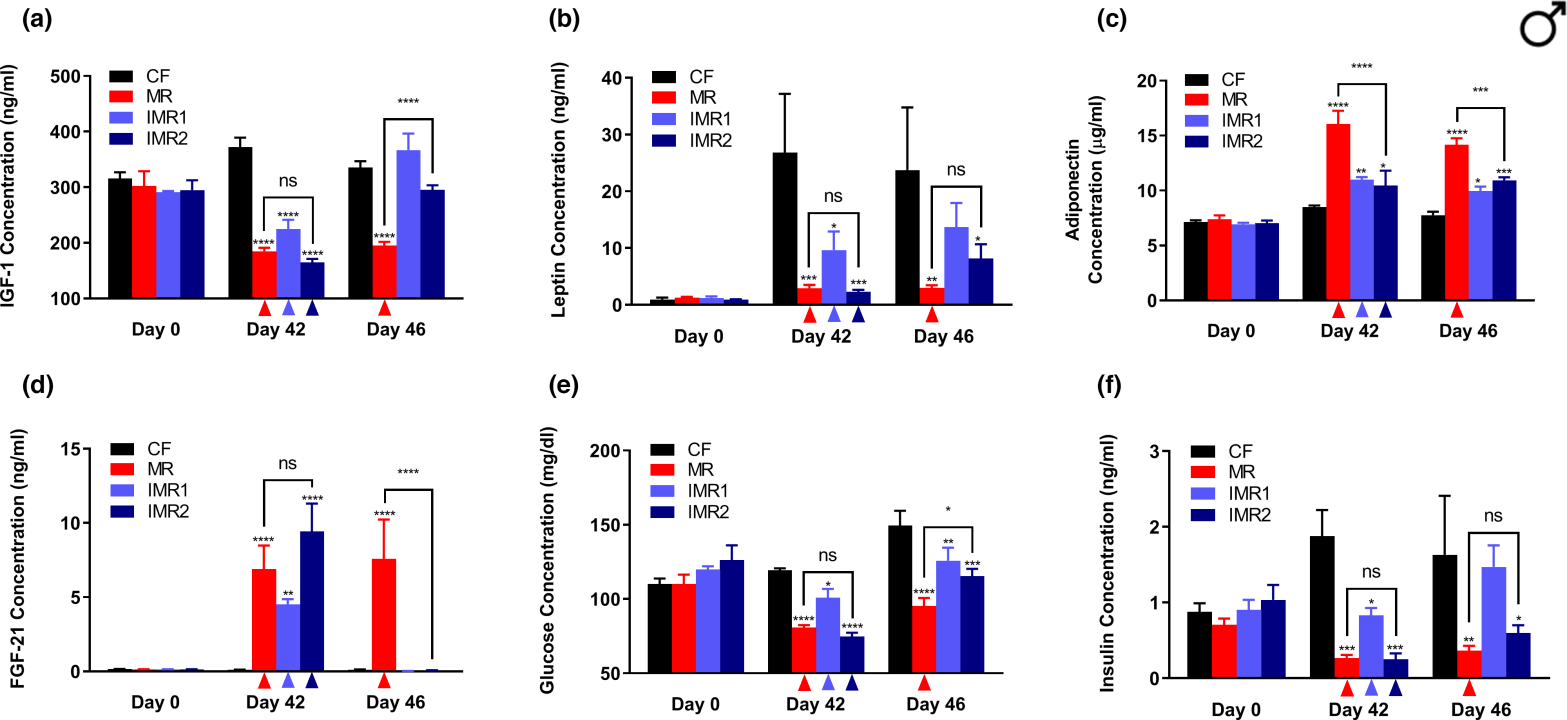
IMR of male mice produces similar beneficial plasma hormone and glycemic changes to continuous MR. Longitudinal comparisons of the plasma concentrations of (a) IGF‐1, (b) leptin, (c) adiponectin, (d) FGF‐21, as well as the blood concentrations of (e) glucose, and (f) insulin for male mice that were control‐fed (CF) or continuously methionine‐restricted (MR), as well as animals subjected to IMR (IMR1) or a more stringent IMR regimen (IMR2). Colored triangles denote that sampling of the indicated groups occurred following a period of MR. Bars denote SEM. Statistically significant differences (as compared with CF values) are indicated (*p < 0.05; **p < 0.01; ***p < 0.001; ****p < 0.0001). Statistically significant differences between MR and IMR2 values are either indicated (*p < 0.05; ***p < 0.001; ****p < 0.0001) or absent (ns). *N* = 4 for all groups

To further explore the extent to which IMR produces the healthspan benefits typically observed for continuous MR, we assessed multiple circulating analytes from male mice at baseline (*i*.*e*., before initiation of the diets), as well as after 42 and 46 days on the diets (with the latter time‐point representing the conclusion of the experiment). For mice on the IMR regimens, the 42 day time‐point followed a period of reduced methionine intake, whereas the 46 day time‐point followed a period of methionine repletion. Plasma samples were analyzed by ELISAs to determine the concentrations of IGF‐1, FGF‐21, leptin, and adiponectin. These analytes were selected because they are not only altered by continuous MR (Ables et al., 2012; Malloy et al., 2006), but have also been implicated in the regulation of metabolism and/or healthspan. Plasma levels of the energy‐regulating hormones adiponectin and leptin are increased and decreased, respectively, by continuous MR, and these changes are likely related to the reduced adiposity of methionine‐restricted animals (Ables et al., 2012; Elshorbagy et al., 2011; Malloy et al., 2006). Levels of the extensively studied hepatokine FGF‐21 are also increased by MR and not only does this hormone play a role in glucose homeostasis (Kharitonenkov et al., 2005), it may also contribute to MR‐dependent lifespan extension through interaction with the IGF‐1 receptor and β‐Klotho (Inagaki et al., 2008; Kurosu et al., 2005). Finally, plasma levels of the anabolic, energy‐regulating hepatokine IGF‐1 are reduced by MR in rodents (Ables et al., 2012; Malloy et al., 2006), and this is apparently sufficient to extend healthspan (Brown‐Borg et al., 2014). That said, we found plasma IGF‐1 levels to be reduced 50% in male mice after 42 days of continuous MR, and saw similar reductions for mice undergoing the IMR regimens (40% and 56%, respectively; Figure 2a). After a subsequent 4 days of methionine repletion, mice undergoing both forms of IMR showed a restoration of IGF‐1 levels to approximately those of control‐fed animals. In addition, we observed that the plasma levels of leptin followed a similar trend to those of IGF‐1, with concentrations in mice undergoing continuous MR failing to increase over time as they did in control‐fed animals (Figure 2b). Both forms of IMR also resulted in the circulating levels of leptin remaining lower than those of control‐fed mice after 42 days on diet (64% and 92%, respectively). Interestingly, plasma leptin levels remained somewhat depressed in males undergoing IMR2 even after a subsequent 4 days of methionine‐replete feeding (66%). In addition, although not as robust as the increases resulting from continuous MR (~85%), modest increases in the plasma levels of adiponectin were produced by both forms of IMR following both a period of limited methionine intake (29% and 23%, respectively), and a period of methionine repletion (28% and 41%, respectively) (Figure 2c). This represents one of the few cases where animals undergoing the otherwise less effective IMR1 regimen received a benefit comparable in magnitude to those undergoing IMR2. Regarding FGF‐21, the levels of this factor in the plasma of male mice fed the experimental diets were as expected, given that FGF‐21 concentrations are known to change dramatically in response to dietary methionine availability (Ables et al., 2012; Perrone et al., 2012). Following a period of limited methionine intake (Day 42), mice undergoing the IMR regimens demonstrated increases in FGF‐21 levels (as compared with controls) that were broadly similar to those of continuously methionine‐restricted mice (IMR1, 42‐fold; IMR2, 87‐fold; MR, 64‐fold; Figure 2d). However, after a period of methionine repletion (Day 46), plasma FGF‐21 levels in animals undergoing both forms of IMR returned to low, control‐fed levels. The final circulating analytes assessed for these animals were glucose and insulin, both of which were found to be significantly lower than control levels on Day 42 in the blood of animals undergoing continuous MR (glucose, 32%; insulin, 86%) and both forms of intermittent MR (glucose, 16% and 37%, respectively; insulin, 56% and 87%, respectively; Figure 2e‐f). On Day 46 (*i*.*e*., following a subsequent 4 days of methionine repletion), glucose levels remained somewhat depressed for mice undergoing IMR (16% and 23%, respectively), whereas reduced insulin levels were only observed for animals undergoing the more stringent of the two IMR regimens (63%). In total, these findings demonstrate that IMR of male mice produces similar metabolic benefits to continuous MR, including altered plasma levels of multiple nutrient‐sensing and longevity‐regulating hormones, as well as improved glycemic control.

### 2.4 Like continuous MR, IMR protects female mice against diet‐induced obesity

**FIGURE 3:**
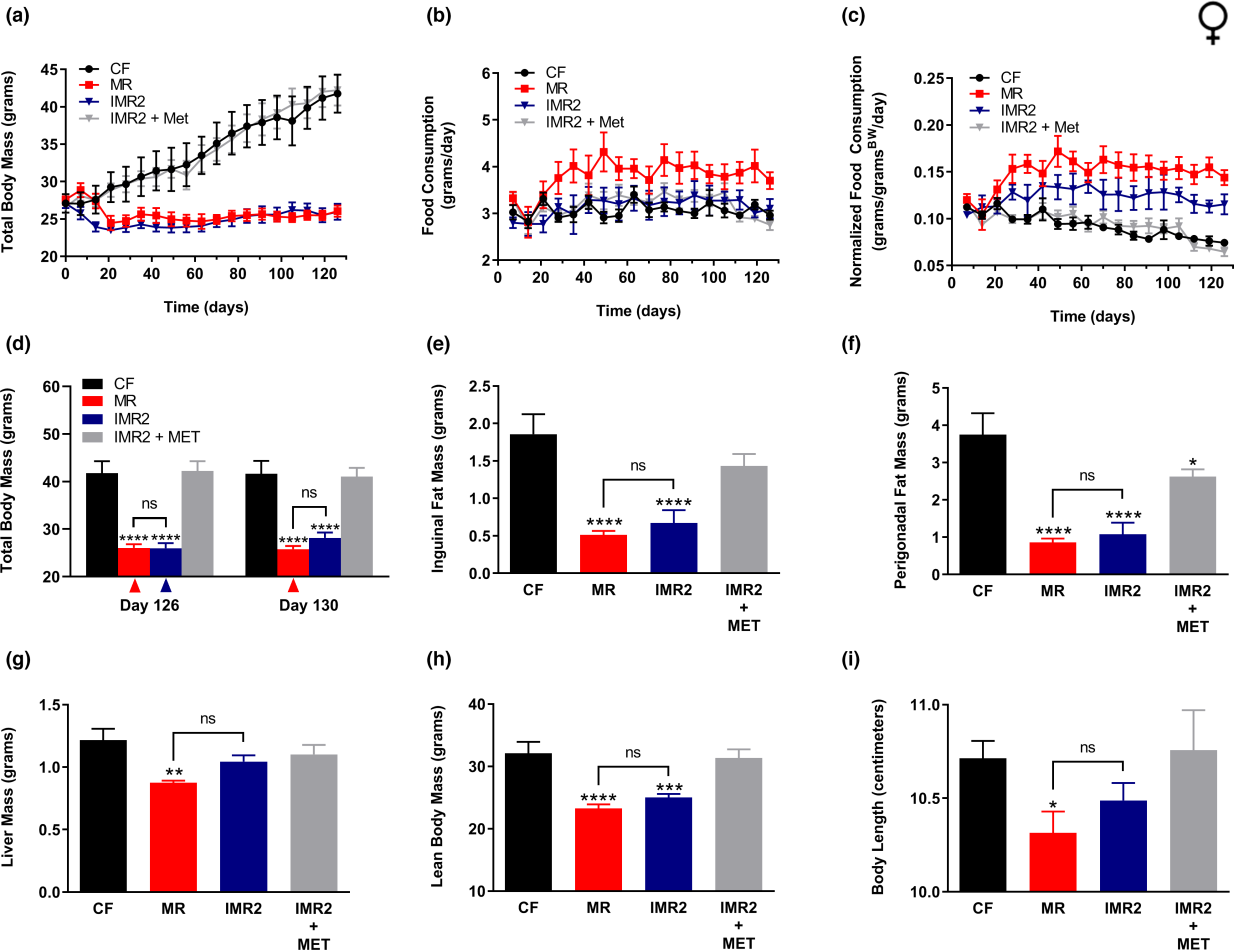
IMR protects female mice against diet‐induced obesity and produces similar healthspan benefits to continuous MR. Comparisons over time of average values for (a) total body mass, (b) food consumption, and (c) food consumption normalized to total body mass for control‐fed (CF; black circles) and continuously methionine‐restricted (MR; red squares) female mice, as well as animals subjected to stringent IMR (IMR2; blue triangles) or animals fed similarly to IMR2 animals but provided methionine in their drinking water (IMR2 + MET; gray triangles). Food consumption graphs (b‐c) show values integrated over 7 days of feeding; expanded graphs are presented in Figure S4. Average values at conclusion of the experiment (~18 weeks) are also shown for (d) total body mass, (e) mass of inguinal fat pads, (f) mass of perigonadal fat pads, (g) liver mass, (h) lean body mass, and (i) body length. For panel d, the colored triangles denote that weighing of the indicated groups occurred following a period of MR. For all panels, bars denote SEM. For panels d‐i, statistically significant differences (as compared with CF values) are indicated (*p < 0.05; **p < 0.01; ***p < 0.001; ****p < 0.0001). No statistically significant differences between MR and IMR2 values were observed (ns). *N* = 8 for all groups

To test whether IMR confers similar benefits to female mice, we made use of an approach like that described above, but with the following differences: (1) of the two IMR regimens, we focused on the more effective IMR2 intervention; (2) an additional negative control was added that featured female mice subjected to IMR2, but supplied with the omitted methionine in their drinking water (IMR2 + MET); (3) animals were fed the experimental diets for 18.5 weeks. These experiments also differed from those performed for males with respect to the age of the animals. As we did previously for a study exploring the healthspan benefits of selenium supplementation (Plummer et al., 2021), we made use of adult females that were older (9 months old) than the males (5 months old) that featured in the above experiments. We favor this approach because we have observed that young female mice remain lean and metabolically healthy even when maintained on a high‐fat diet (not shown). In any case, as above, body mass and food consumption were measured repeatedly until the end of the experiment (Figure 3a‐d, Figure S4), at which point fat pads and livers were surgically resected and weighed (Figure 3e‐g). Additional body condition measurements (*i*.*e*., lean body mass and body length) were also made at this time (Figure 3h‐i). As expected, we found that animals fed the control diet experienced a dramatic increase in total body mass (Figure 3a,d), as well as a significant accumulation of adiposity (Figure 3e‐f). Conversely, both continuous MR and the IMR2 regimen resulted in animals remaining lean over the course of the experiment (Figure 3a,d). These interventions also resulted in reduced accumulation of both inguinal (MR, 72%; IMR2, 64%) and perigonadal (MR, 77%; IMR2, 71%) adiposity, as compared with controls (Figure 3e‐f). In fact, the same effect of the IMR2 regimen on adiposity was observed even when fat pad mass was normalized to lean body mass (Figure S5a‐b). Similar to the case for males, liver mass was not affected by IMR (Figure 3g, Figure S5c). However, female mice subjected to this intervention did show somewhat less lean body mass than control‐fed animals (22%; Figure 3h). In contrast, the average overall body length of female mice undergoing the IMR2 regimen was not significantly different from that of controls (Figure 3i). As expected, continuously methionine‐restricted females experienced multiple forms of growth inhibition, as they had 27% less lean body mass than controls (Figure 3h) and were also 4% shorter (Figure 3i). Finally, as was true for the experiments featuring males, the benefits received by intermittently methionine‐restricted female mice were not due to reduced calorie intake, as we found their food consumption to be at least that of control‐fed animals, regardless of whether or not these values were normalized for body mass (Figure 3b‐c, Figure S5). Taken together, our experiments clearly demonstrate that, like continuous MR, IMR protects both male and female mice against diet‐induced obesity.

### 2.5 Like continuous MR, IMR confers beneficial metabolic and plasma hormone changes to female mice

**FIGURE 4:**
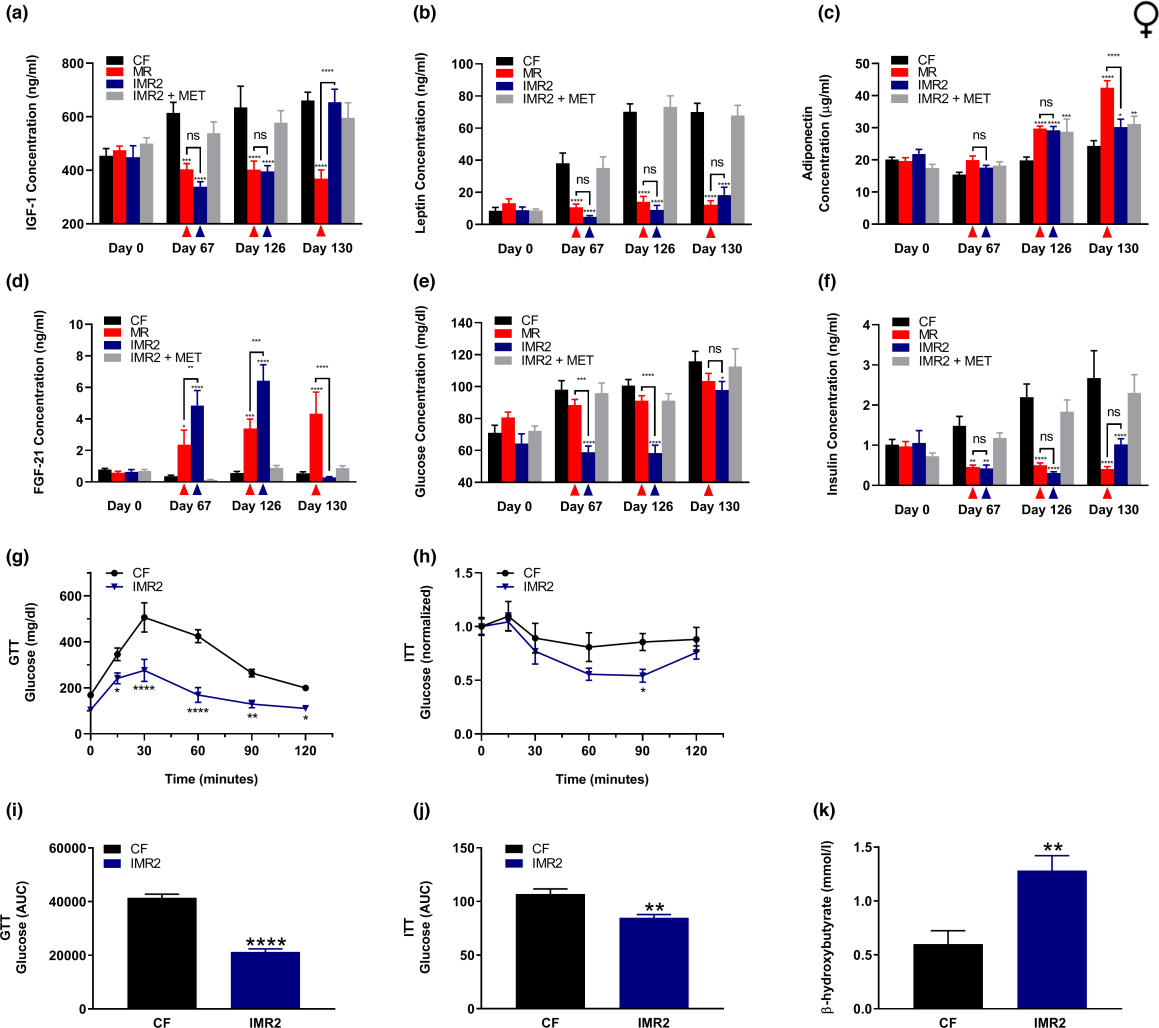
IMR of female mice produces similar beneficial plasma hormone and glycemic changes to continuous MR. Longitudinal comparisons of the plasma concentrations of (a) IGF‐1, (b) leptin, (c) adiponectin, (d) FGF‐21, as well as the blood concentrations of (e) glucose, and (f) insulin for female mice that were control‐fed (CF), continuously methionine‐restricted (MR), subjected to stringent IMR (IMR2), or fed similarly to IMR2 animals but provided methionine in their drinking water (IMR2 + MET). Colored triangles denote that sampling of the indicated groups occurred following a period of MR. Metabolic tolerance tests (glucose, GTT; insulin, ITT) were performed for female mice that were either control‐fed or subjected to stringent IMR (g‐h), as well as area‐under‐the‐curve (AUC) analyses of the resulting data (i‐j), and determination of blood concentrations of β‐hydroxybutyrate for animals subjected to control or IMR feeding (k). For all panels, bars denote SEM. Statistically significant differences (as compared with CF values) are indicated (*p < 0.05; **p < 0.01; ***p < 0.001; ****p < 0.0001). For panels a‐f, statistically significant differences between MR and IMR2 values are either indicated (**p < 0.01; ***p < 0.001; ****p < 0.0001) or absent (ns). For panels A‐F, *N* = 8 for all groups; for panels g‐k, *N* = 6 for all groups

To further our analyses of the benefits of IMR to female mice, we also assessed their circulating levels of IGF‐1, leptin, adiponectin, FGF‐21, glucose, and insulin. Overall, the results mirrored what was observed for intermittently methionine‐restricted males. For example, after both 67 and 126 days of feeding, the average circulating IGF‐1 levels of intermittently methionine‐restricted females were approximately 40% lower than those of control‐fed littermates, a relative decrease similar to that of continuously methionine‐restricted animals (Figure 4a). However, on Day 130, after 4 subsequent days of methionine repletion, mice undergoing the IMR2 regimen showed IGF‐1 levels equivalent to those of controls. With respect to leptin, we observed a significant and persistent reduction in the levels of this hormone in intermittently methionine‐restricted females as compared with control‐fed littermates, remaining 74% lower than control levels even after a period of methionine repletion (Day 130; Figure 4b). For adiponectin levels, we observed a relative increase (47%) on Day 126 for animals undergoing IMR2; following 4 days of methionine repletion, these levels lowered yet remained higher than controls (24%; Figure 4c). As expected, FGF‐21 also showed a dramatic, but transient, increase in the plasma of intermittently methionine‐restricted female mice, with values peaking at 11‐fold greater than those of control‐fed animals on Day 126 before returning to control levels after the methionine‐replete period (Day 130; Figure 4d).

**FIGURE 5:**
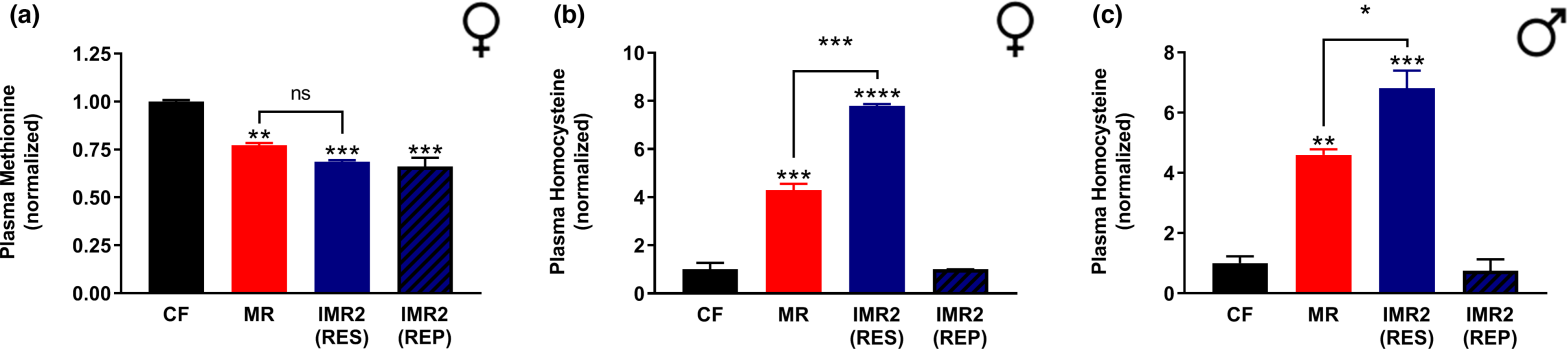
Plasma levels of sulfur amino acids are similar for mice subjected to IMR and continuous MR. Comparisons of the relative plasma levels of (a) methionine and (b‐c) homocysteine are shown for female (a‐b) and male (c) mice that were control‐fed (CF), continuously methionine‐restricted (MR), or subjected to stringent IMR (IMR2). IMR2 values represent sampling following either a period of methionine restriction (RES) or methionine repletion (REP). Prior to sampling, animals were fed the indicated diets for ~18 weeks (a‐b) or ~6 weeks (c). Bars denote SEM. Statistically significant differences (as compared with the corresponding CF values) are indicated (**p < 0.01; ***p < 0.001; ****p < 0.0001). Statistically significant differences between MR and IMR2 (RES) values are either indicated (*p < 0.05; ***p < 0.001) or absent (ns). For panels a‐b, N=8 for all groups; for panel c, *N* = 4 for all groups

Like males, female mice also demonstrated improved glycemic control in response to IMR, evidenced by relative reductions in the levels of circulating glucose and insulin (Figure 4e‐f). Indeed, intermittently methionine‐restricted female mice had lower circulating glucose levels than both control‐fed and continuously methionine‐restricted animals at all time‐points of the feeding study (Figure 4e). Interestingly, we have previously observed that female mice only show reduced insulin (but not glucose) levels in response to continuous MR (Plummer et al., 2021), a finding that is reproduced in the current study (Figure 4e). It is therefore notable that females undergoing the IMR2 regimen maintained relatively low blood glucose levels, the same as males undergoing either intermittent or continuous MR (Figure 2e). With respect to circulating insulin levels, IMR of female mice again resulted in lower levels than those of control animals (Figure 4f). These depressed levels were apparent following periods of both reduced methionine intake (Day 67, 71%; Day 126, 86%) and methionine repletion (Day 130, 62%). To further explore the effects of IMR on glucose metabolism, we performed metabolic tolerance tests on 12‐month‐old female mice that were either control‐fed or intermittently methionine‐restricted for 3 weeks (glucose tolerance; Figure 4g) or 5 weeks (insulin tolerance; Figure 4h). For animals subjected to IMR2, testing coincided with a period of reduced methionine intake. Given that continuous MR has been previously demonstrated to effectively prevent the glucose intolerance and insulin insensitivity otherwise experienced by mice maintained on a high‐fat diet (Ables et al., 2012), we reasoned that IMR would result in similar protection. As expected, and consistent with the effects of IMR on the circulating levels of both glucose and insulin, mice undergoing this intervention were significantly more glucose‐tolerant and insulin‐sensitive than control‐fed littermates (Figure 4g‐j).

Given that continuous MR has been shown to elevate the circulating levels of the beneficial ketone body β‐hydroxybutyrate (Malloy et al., 2013), we also tested whether IMR shared this capability. As expected, we found the 12‐month‐old female mice subjected to the IMR2 regimen for 3 weeks possessed elevated blood levels of β‐hydroxybutyrate as compared with control‐fed littermates (Figure 4k). In total, our experiments demonstrate that IMR not only confers to female mice similar beneficial changes in the levels of multiple circulating factors as continuous MR, but also that the novel intervention may actually be more effective than its continuous counterpart at maintaining glucose homeostasis in the face of a high‐fat diet.

### 2.6 IMR and continuous MR similarly alter the plasma levels of multiple sulfur‐containing amino acids

As a final test of the similarities of continuous MR and IMR, we explored the effects of these interventions on the circulating levels of methionine and homocysteine. Reduced plasma levels of methionine are often, but not always, observed in response to reduced methionine intake (Ables et al., 2015; Elshorbagy et al., 2010; Olsen et al., 2018). Somewhat paradoxically, the most robust effect of continuous MR on the circulating levels of sulfur‐containing amino acids is an increase in the levels of homocysteine. As expected, we observed an approximately 70% reduction in the plasma levels of methionine in both continuously and intermittently methionine‐restricted female mice as compared with control‐fed animals (Figure 5a). A similar reduction was observed for males (not shown). Interestingly, in both cases, these reductions were observed for animals undergoing IMR after both the methionine‐restricted and methionine‐replete periods (Figure 5a; not shown). As for homocysteine, both continuous and intermittent MR resulted in relative increases in the plasma levels of this amino acid in both male and female mice (Figure 5b‐c). Following the methionine‐restricted period of the IMR regimen, homocysteine levels were ~7‐fold higher than those of control animals, whereas no difference was observed following the methionine‐replete period. Taken together, the shared effects of continuous MR and IMR on the circulating levels of both methionine and homocysteine provide yet additional evidence that these interventions are functionally similar.

### 2.7 Alternate‐day IMR is similarly effective to contiguous‐day IMR

**FIGURE 6:**
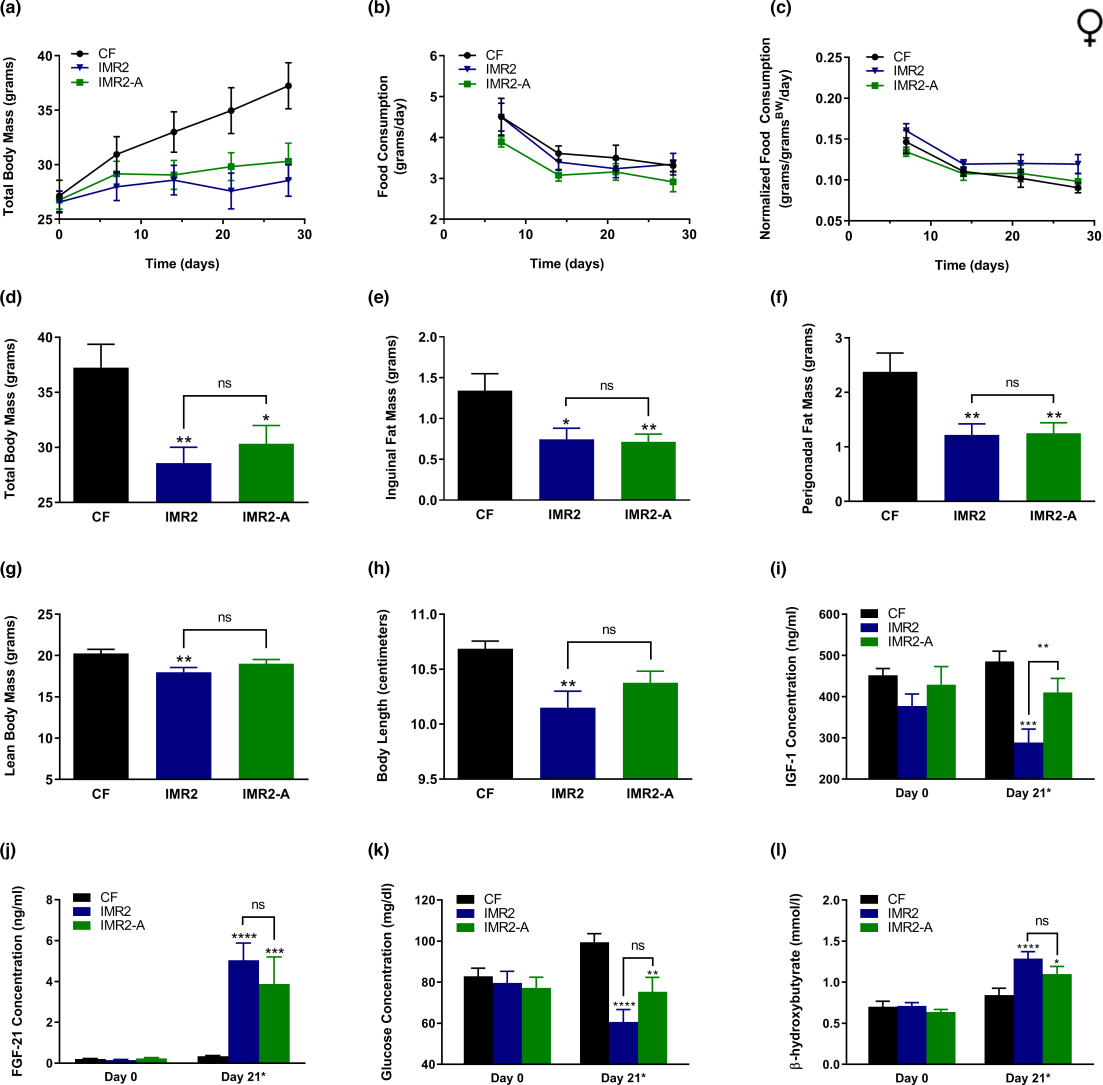
Contiguous‐day IMR and alternate‐day IMR confer similar protection against diet‐induced obesity and similar beneficial plasma hormone and glycemic changes. Comparisons over time of average values for (a) total body mass, (b) food consumption, and (c) food consumption normalized to total body mass for control‐fed female mice (CF; black circles), as well as animals subjected to stringent IMR (IMR2; blue triangles) or fed a modified IMR2 regimen featuring alternating days of methionine restriction and repletion (IMR2‐A; green squares). Food consumption graphs (b‐c) show values integrated over 7 days of feeding; expanded graphs are presented in Figure S6. Average values at conclusion of the experiment (4 weeks) are also shown for (d) total body mass, (e) mass of inguinal fat pads, (f) mass of perigonadal fat pads, (g) lean body mass, and (h) body length. Longitudinal comparisons of the plasma concentrations of (i) IGF‐1 and (j) FGF‐21 were also performed, as well as determination of the blood concentrations of (k) glucose and (l) β‐hydroxybutyrate; control‐fed mice and animals subjected to IMR2 were sampled at baseline and on Day 21, whereas animals undergoing the IMR2‐A regimen were sampled at baseline and on Day 19 (Day 21*). For all panels, bars denote SEM. For panels d‐l, statistically significant differences (as compared with CF values) are indicated (*p < 0.05; **p < 0.01; ***p < 0.001; ****p < 0.0001); statistically significant differences between IMR2 and IMR2‐A values are either indicated (**p < 0.01) or absent (ns). *N* = 8 for all groups

Finally, we sought to assess the flexibility of the IMR regimen by determining whether 3 contiguous days of reduced methionine intake are required for its effectiveness. Toward this end, we developed a variant of stringent IMR (IMR2‐A) that alternates daily between methionine‐restricted and methionine‐replete feeding for 6 days, before concluding each week with a final day of methionine repletion (Figure S1). Thus, in the IMR2‐A feeding scheme, 3 days of reduced methionine intake are spread across a 5‐day period, rather than being contiguous (as for IMR2). Importantly, the time‐integrated concentrations of methionine availability (0.49%) are identical for both regimens. Adult female mice (9+ months old) were fed the control diet and the IMR2 and IMR2‐A regimens for a period of 4 weeks, at the end of which their body condition (Figure 6a,d‐h) and food consumption (Figure 6b‐c, Figure S6) were assessed. In addition, the relative levels of multiple circulating markers of metabolism were determined (Figure 6i‐l). We found IMR2 and IMR2‐A to be similarly effective at protecting mice against the overall weight gain (Figure 6a,d) and inguinal and perigonadal adiposity (Figure 6e‐f) experienced by control‐fed animals. The latter finding was true whether or not fat mass values were normalized to lean body mass (Figure S7). We also found food consumption between all three feeding groups to be essentially equivalent (Figure 6b‐c, Figure S5), confirming that the observed benefits were not due to calorie restriction. As observed previously, the IMR2 regimen resulted in modest impairments in lean body mass and overall body length as compared with control‐fed littermates (Figure 6g‐h). However, similar measurements for mice subjected to IMR2‐A were not found to be significantly different from either control values or IMR2 values. That said, analysis of circulating factors (*i*.*e*., IGF‐1 and glucose) in these animals was technically challenging owing to differences in the timing of the restricted/replete periods in the IMR2 and IMR2‐A regimens. While control‐fed mice and littermates undergoing IMR2 were sampled on Day 21 (the last of 3 contiguous days of restriction for animals undergoing IMR2), the most equivalent time‐point for animals undergoing IMR2‐A (*i*.*e*., the last day of a more temporally expanded period of reduced methionine intake) occurred 2 days prior on Day 19. In addition, given that animals subjected to IMR2‐A do not experience an uninterrupted period of reduced methionine intake, as do IMR2‐fed littermates, it would be expected that the effects of IMR2‐A on the levels of circulating factors (measured on Day 19) might be somewhat less robust than those of IMR2 (measured on Day 21). Indeed, this was the case for measurements of IGF‐1, FGF‐21, glucose, and β‐hydroxybutyrate (Figure 6i‐l). Nevertheless, the slightly attenuated values observed for FGF‐21, glucose, and β‐hydroxybutyrate in mice undergoing IMR2‐A were not found to be significantly different from those of IMR2‐fed littermates. As a result, we consider it likely that the time‐integrated blood concentrations of these factors were similar between animals subjected to IMR2 or IMR2‐A, consistent with the near identical protection against diet‐induced obesity conferred by these interventions (Figure 6a,d‐f,k, Figure S7). Together, these findings indicate that IMR is capable of producing robust healthspan benefits, regardless of the specific timing of the methionine‐restricted periods.

## 3 DISCUSSION

To the best of our knowledge, we show for the first time that IMR confers to male and female mice the beneficial metabolic effects previously reported for continuous MR. That is, as compared with control‐fed mice, animals undergoing the more stringent of two IMR regimens (IMR2) benefit from reduced adipose tissue accumulation, protection against hepatosteatosis, improved glucose homeostasis, and altered circulating levels of IGF‐1, FGF‐21, leptin, and adiponectin. Despite a much shorter interventional period than continuous MR (only 3 days per week), not only is IMR capable of producing similar health benefits, but other aspects of this intervention are actually superior to classical MR. For example, while we have repeatedly observed that female mice have a somewhat limited response to continuous MR, with this intervention only partially improving their glucose homeostasis in the context of a high‐fat diet (Plummer et al., 2021), IMR results in consistently low levels of both circulating glucose and insulin in both males and females. In addition, we find that the IMR regimen is fairly flexible, as similar healthspan benefits are produced whether the period of restriction is continuous (*i*.*e*., 3 contiguous days; IMR2) or interspersed with days of control feeding, as in the case of IMR2‐A.

In terms of how, mechanistically, IMR engenders such dramatic healthspan‐promoting effects despite its short interventional period, we first considered the calculated methionine intake of mice undergoing the experimental regimens. When integrated over a week of feeding, the concentrations of methionine in the dietary regimens should be as follows: 0.86% (CF), 0.12% (MR), 0.54% (IMR1), and 0.49% (IMR2 and IMR2‐A). Consequently, if one were to assume that the extent of the benefits of MR are inversely proportional to methionine intake, then animals undergoing IMR2 would, at best, show physiological effects intermediate to control‐fed and methionine‐restricted animals (*i*.*e*., 0.86% > 0.49% > 0.12%). Similarly, one would also expect that animals undergoing the IMR1 and IMR2 regimens would be physiologically and metabolically similar, as the time‐integrated methionine contents of their diets differ by only 0.05%. As we found neither of these suppositions to be true, we instead favor the explanation that the benefits of MR (both continuous and intermittent) result from a threshold effect; in this case, the reduction of circulating glucose and insulin levels below some critical threshold that, in turn, results in significant and persistent changes in the GH/IGF‐1 signaling axis. In support of such an idea, we found that, during methionine‐restricted periods, circulating glucose was maintained by the IMR2 regimen at levels at least as low as those of continuously methionine‐restricted animals. While the low glucose levels resulting from IMR2 and continuous MR were comparable for males, the levels in females were far more responsive to the IMR2 regimen than to continuous MR. That continuous MR fails to protect female mice against increasing blood glucose levels (as it does for males) could be (1) a gender‐specific effect, or (2) due to the advanced age of the females used for these studies (9 months old vs 5 months old for males). Previous work from researchers at the Orentreich Foundation and others supports the latter explanation, having found that rodents require less methionine as they age, and consequently, that older rodents show an attenuated response to typical levels of MR (Ishibashi & Kametaka, 1977; Nichenametla et al., 2020; Shin et al., 1994). Thus, the effective maintenance of glucose homeostasis in females undergoing the IMR2 regimen is likely due to the brief periods of stringent MR that they experience. In any case, the robustness of the response to IMR is consistent with a previous study that found that a period of stringent MR confers significant metabolic benefits to obese mice (Yu et al., 2018). It is also interesting to note that, in the current study, the effect of the IMR2 regimen on circulating glucose was relatively persistent, as even after 4 days of methionine‐replete feeding, the blood glucose levels of animals undergoing this intervention often remained lower than those of control‐fed littermates. Such normalization of glucose homeostasis is likely to be a function of altered carbon metabolism, as has been reported for mice, humans, and even yeast subjected to methionine‐restricted conditions (Malloy et al., 2013; Plaisance et al., 2011; Plummer & Johnson, 2019). In turn, as a consequence of maintaining persistently low circulating glucose and insulin levels, IMR reduces IGF‐1 hormone levels, thereby producing additional healthspan benefits. Not only have low IGF‐1 levels been previously reported for continuously methionine‐restricted rodents (Ables et al., 2012; Malloy et al., 2006; Miller et al., 2005), but also a multitude of other healthspan‐extending interventions are known to both alter carbon metabolism and decrease IGF‐1 signaling, including CR, intermittent fasting, the ketogenic diet, protein restriction, and exercise (Boden et al., 2005; Breese et al., 1991; Dunn et al., 1997; Fontana et al., 2008; Fraser et al., 2000; Rahmani et al., 2019; Scarth, 2006). That continuous MR fails to extend the lifespan of long‐lived dwarf mice that already have constitutively low GH/IGF‐1 levels (Brown‐Borg et al., 2014) suggests that MR extends healthspan by decreasing IGF‐1 signaling. This is further supported by the fact that GH‐overexpressing transgenic mice have an impaired response to continuous MR (Brown‐Borg et al., 2018).

Regarding how the timing of IGF‐1 reductions influences the healthspan benefits that result, it was previously reported that a transient period of food restriction during the first 20 days of life results in low serum IGF‐1 levels and extends the median and maximal survival of mice (Sun et al., 2009). The authors suggested that a brief period of food restriction early in development may produce life‐long effects through stable reprogramming of gene expression. Indeed, in a subsequent study, the same group found that mice subjected to such early‐life restriction remain leaner than controls and also have better glucose homeostasis, even 22 months after the interventional period (Sadagurski et al., 2014). The authors also demonstrated that activation of the GH/IGF‐1 signaling axis for a period of 6 weeks *via* regular injections of GH in young dwarf mice (starting at 2 weeks of age) substantially limits the lifespan of these otherwise long‐lived animals (Panici et al., 2010). It is clear from such studies that not only can a transient reduction in circulating IGF‐1 during development confer life‐long healthspan benefits, but also a transient restoration of IGF‐1 activity in dwarf mice can abrogate such benefits. Regarding effects in adult animals, Sun et al. also explored the efficacy of continuous MR initiated at mid‐life (12 months of age) and found that this intervention results in a significant extension of lifespan (Sun et al., 2009). Superficially, this result may seem to be at odds with that of Panici et al., which found that reduced IGF‐1 signaling following an early period of GH/IGF‐1 activity is insufficient to support lifespan extension. However, the differing outcomes of these studies may be due, at least in part, to their different genetic contexts (*i*.*e*., wild‐type hybrid mice vs *Prop1^df^*/ *Prop1^df^* hypopituitary dwarf mice). When all of the above studies are taken together, the extended lifespan observed by Sun et al. for 12‐month‐old wild‐type mice subjected to MR is probably best explained by (1) the fact that mice have persistently high circulating levels of IGF‐1, even in mid‐life (Ashpole et al., 2017), and (2) the likelihood that any intervention that reduces these high levels or otherwise impairs IGF‐1 signaling, even transiently, will improve healthspan. Thus, it is possible that, like continuous MR, IMR will extend the overall survival of mice. While future experiments will reveal whether this is the case, it is clear from the current study that IMR produces a multitude of metabolic health benefits previously reported for continuous MR. Further, the more practicable diet (which necessitates an interventional period of only 3 days per week) confers to female mice an improvement in glucose homeostasis that continuous MR fails to provide. As a result, we consider IMR to not only be a superior alternative to the classical intervention, but also an ideal candidate for translation to humans in order to reduce the burden of both metabolic and age‐related disease.

## 4 EXPERIMENTAL PROCEDURES

### 4.1 Animal care and feeding

All animal studies were approved by the Institutional Animal Care and Use Committee (IACUC) of the Orentreich Foundation for the Advancement of Science, Inc. (Permit Number: 0511MB). C57BL/6J mice (Stock number 000664) were purchased from the Jackson Laboratories (Bar Harbor, ME) and housed in a conventional animal facility maintained at 20±2°C, 50±10% relative humidity, with a 12 h/12 h light/dark photoperiod. Food and water were provided *ad libitum*. Upon arrival, mice were acclimatized for up to one week and fed Purina Lab Chow 5001 (Ralston Purina, Co.; St. Louis, MO). For feeding studies, mice were given one of three isocaloric (5.3 kcal/gm) high‐fat diets, comprising 12% kcal protein, 31% kcal carbohydrate, and 57% kcal fat (Research Diets; New Brunswick, NJ). Essentially, these diets were formulated as follows, 1) 0.86% methionine (control), 2) 0.12% methionine (methionine‐restricted), and 3) 0% methionine (methionine‐free), which were used to subject mice to control‐fed, continuously methionine‐restricted, and intermittently methionine‐restricted dietary regimens. Full details concerning diet compositions and feeding regimens are given in Table S1 and Figure S1, respectively. In addition, detailed methods for animal monitoring, tissue collection, liver histological analyses, metabolic tolerance tests, plasma amino acid analyses, and statistical analyses are provided in Supporting Information. A table of all key resources is also provided (Table S2).

### 4.2 Blood hormone tests

Enzyme‐linked immunosorbent assay (ELISA) kits were obtained commercially and used to measure plasma levels of IGF‐1 (R&D Systems; Minneapolis, MN), adiponectin (R&D Systems), FGF‐21 (Millipore Corp.; Billerica, MA), leptin (R&D Systems), and insulin (ALPCO Diagnostics; Salem, NH). All tests were performed according to the manufacturers’ recommendations and measured using a Molecular Devices SpectraMax M5 Microplate Reader (Molecular Devices LLC; San Jose, CA). Two technical replicates were performed for each sample.

## AUTHOR CONTRIBUTIONS

JJ designed the study, assisted with sample collection, analyzed the data, and wrote the manuscript. JP performed all the experiments (with the exception of the liver histology and microscopy), analyzed the data, and read and approved the manuscript.

## CONFLICT OF INTEREST

None declared.

## Supporting information

Fig S1

Fig S2

Fig S3

Fig S4

Fig S5

Fig S6

Fig S7

Supplementary Material

## Data Availability

All data associated with this study are present in the manuscript or as supplementary information.
