# Supplementary figures and images for: Intermittent methionine restriction reduces IGF‐1 levels and produces similar healthspan benefits to continuous methionine restriction
Source: Aging Cell. 2022 May 15;21(6):e13629. doi: 10.1111/acel.13629 (PMC9197402; doi:10.1111/acel.13629)

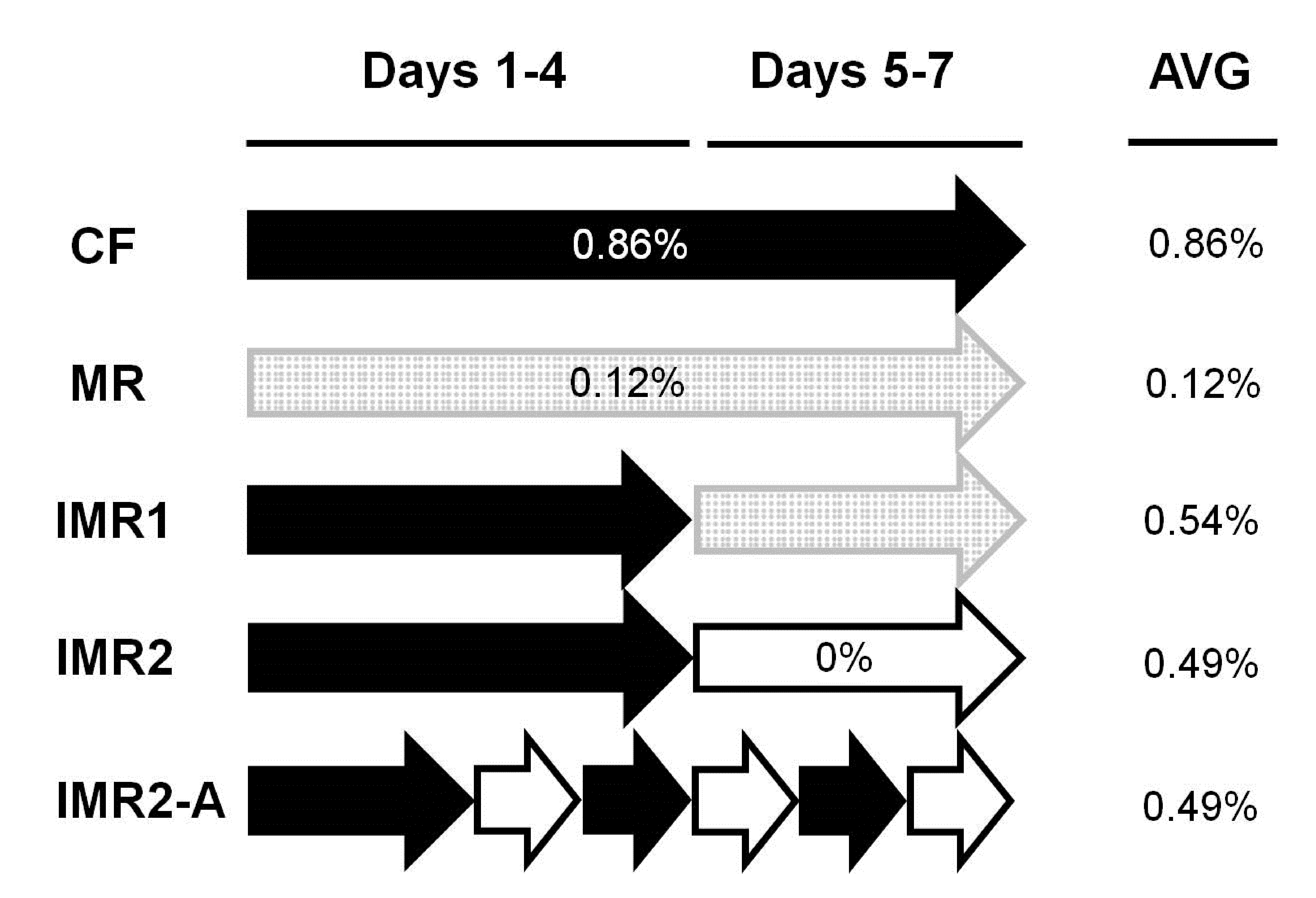

Supplement: Supplementary file 1 — Fig S1 [file ACEL-21-e13629-s008.tif]

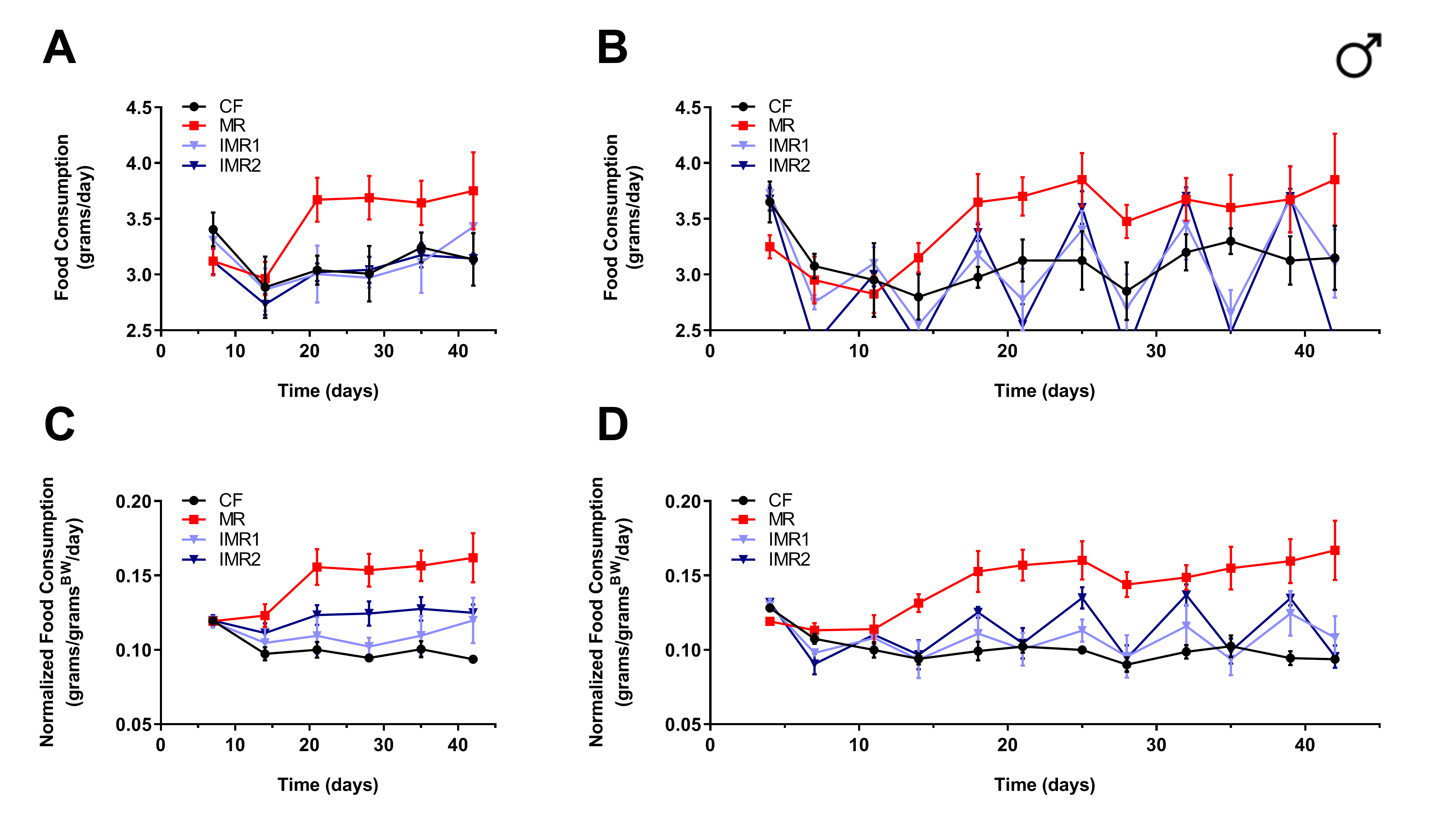

Supplement: Supplementary file 2 — Fig S2 [file ACEL-21-e13629-s005.tif]

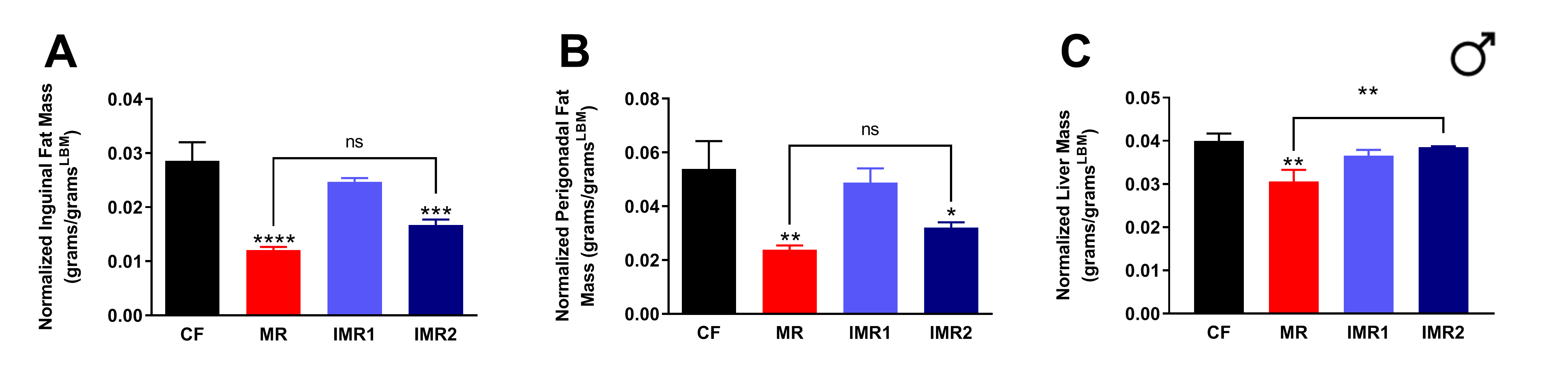

Supplement: Supplementary file 3 — Fig S3 [file ACEL-21-e13629-s001.tif]

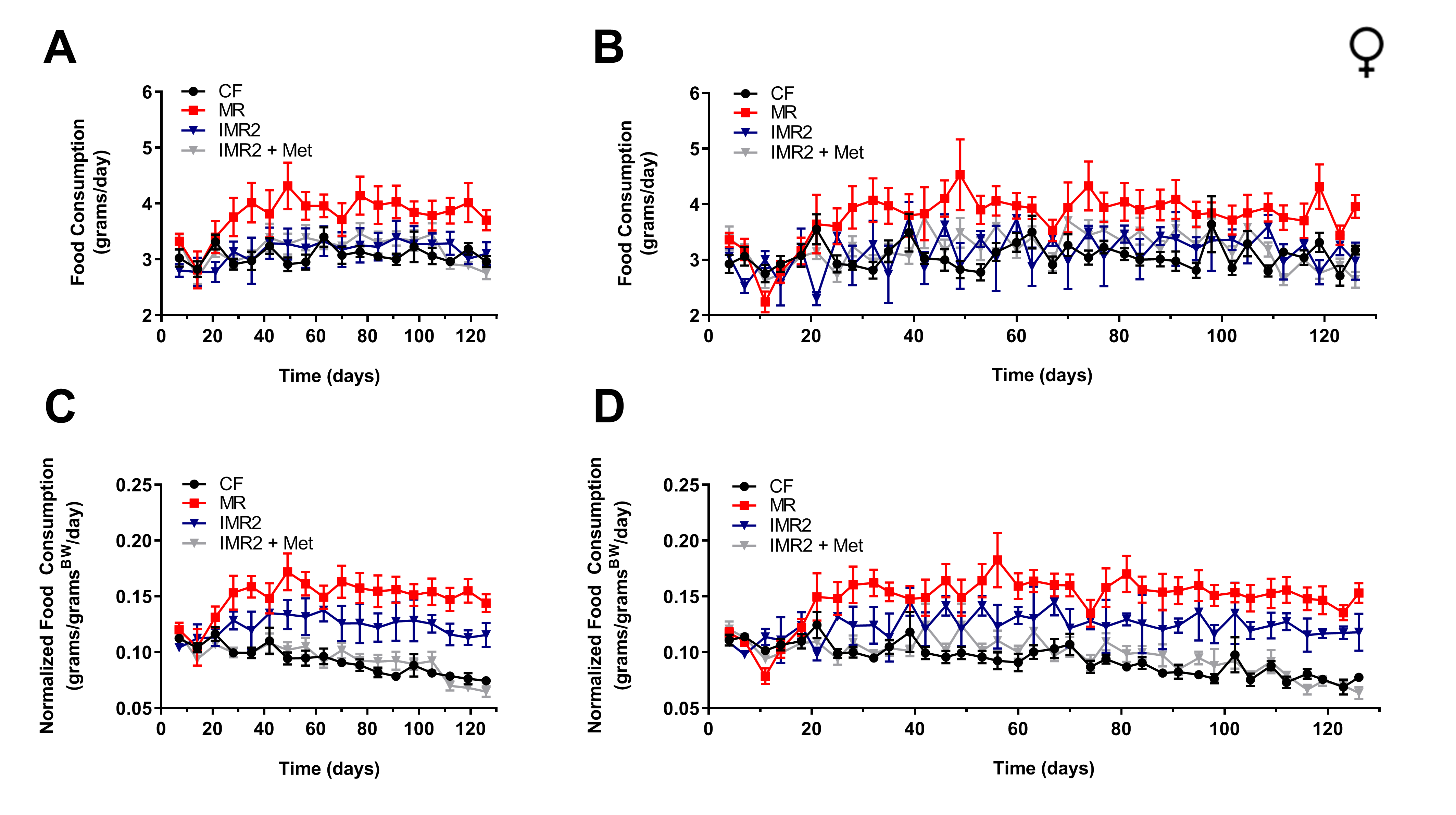

Supplement: Supplementary file 4 — Fig S4 [file ACEL-21-e13629-s002.tif]

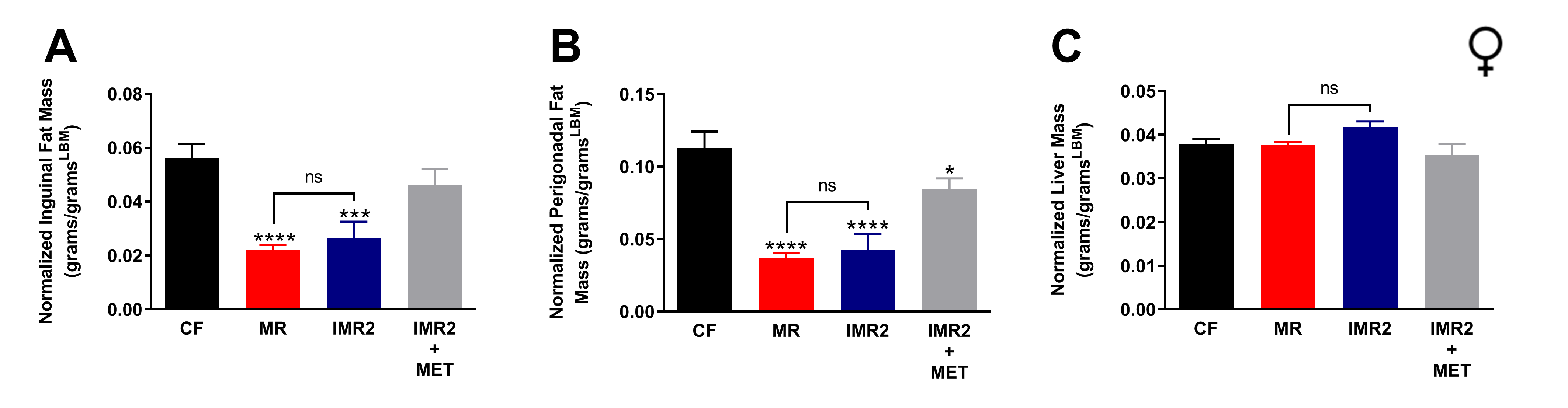

Supplement: Supplementary file 5 — Fig S5 [file ACEL-21-e13629-s007.tif]

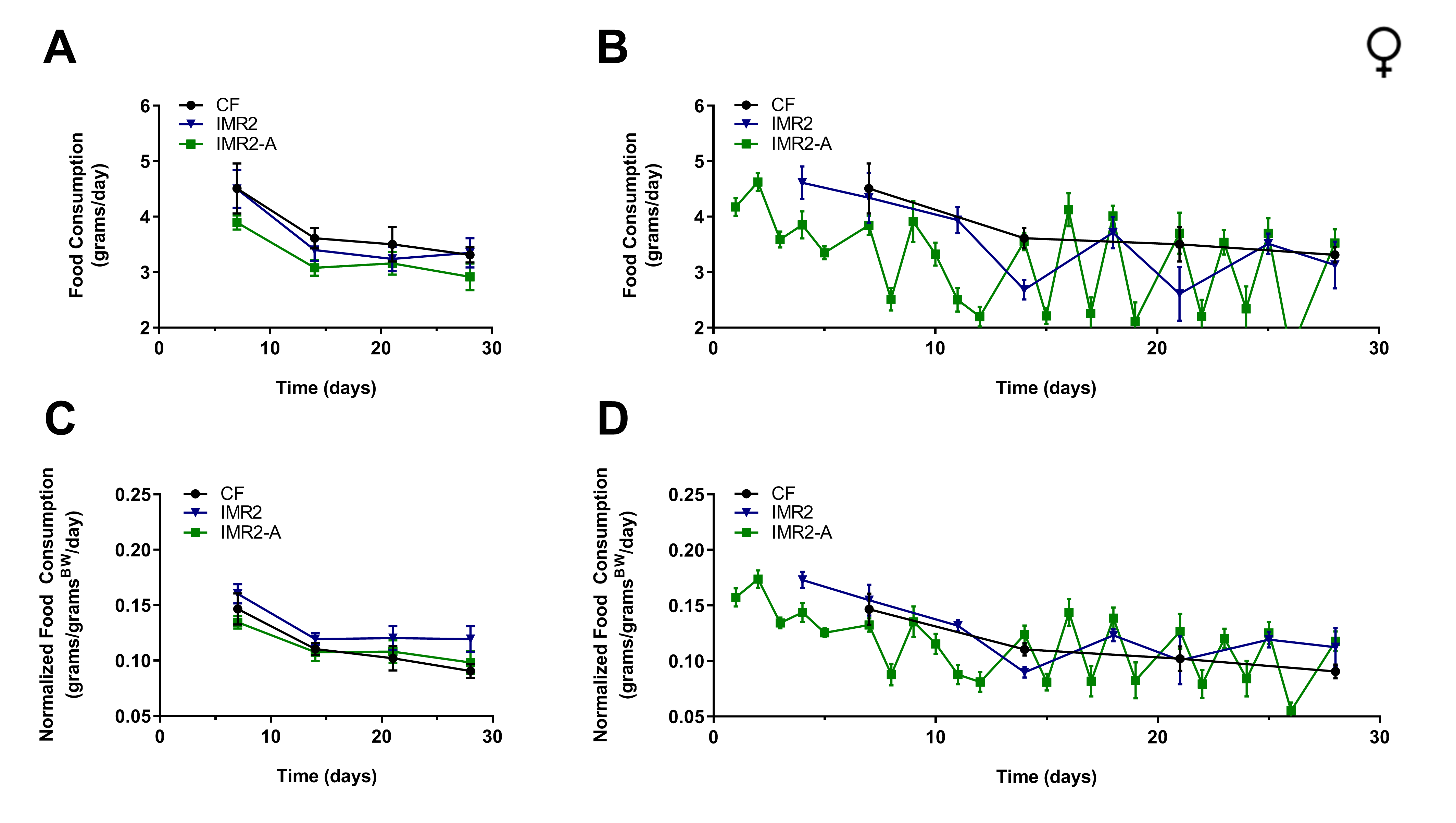

Supplement: Supplementary file 6 — Fig S6 [file ACEL-21-e13629-s003.tif]

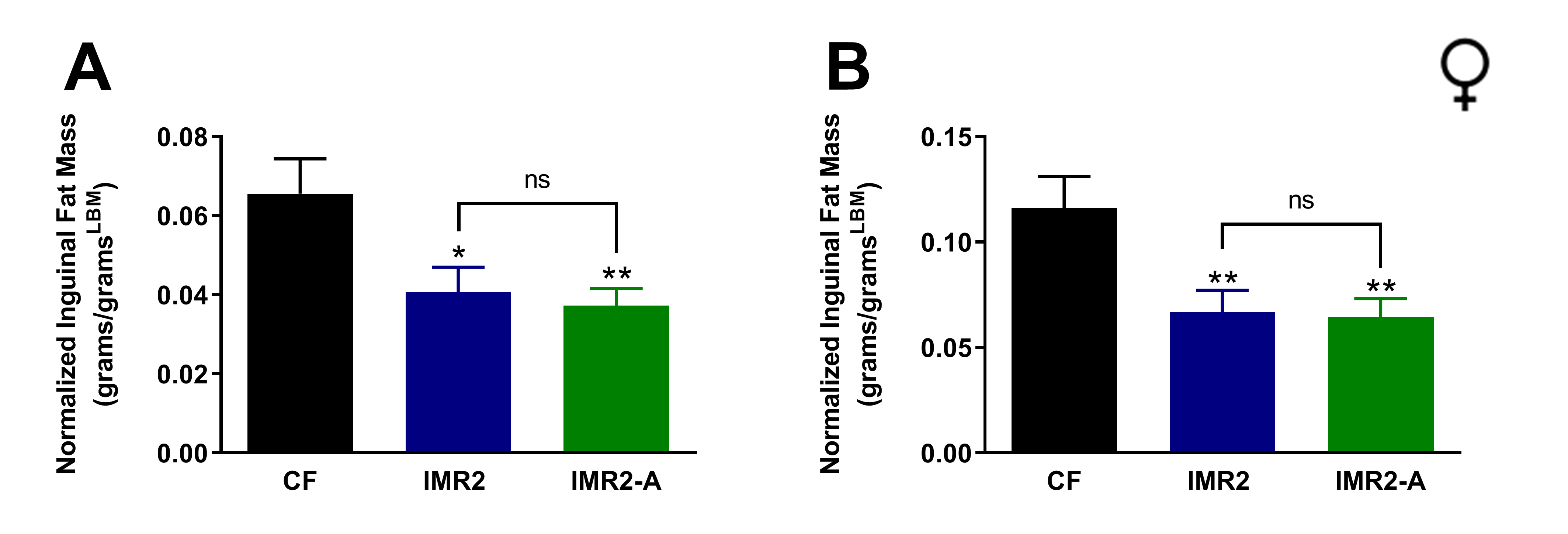

Supplement: Supplementary file 7 — Fig S7 [file ACEL-21-e13629-s006.tif]
