# Supplementary material for: Intermittent methionine restriction reduces IGF‐1 levels and produces similar healthspan benefits to continuous methionine restriction
Source: Aging Cell. 2022 May 15;21(6):e13629. doi: 10.1111/acel.13629 (PMC9197402; doi:10.1111/acel.13629)
Supplement: Supplementary file 8 — Supplementary Material [file ACEL-21-e13629-s004.docx]

**SUPPORTING INFORMATION**

**EXPERIMENTAL PROCEDURES**

**Animal Monitoring and Tissue Collection**

Mice were randomly assigned to each of the diet groups such that each group had a similar average body mass (*i.e.*, weight-matched). Once assigned, no animals (or samples resulting therefrom) were removed from the study. Body mass and food consumption were monitored once a week for the duration of the study. Prior to blood collection, animals were fasted for 4 hrs to establish physiological baselines. Blood was then collected from the retro-orbital plexus, processed using EDTA-K2-coated blood collection tubes (Milian Dutscher Group; Geneva, Switzerland), and the resulting plasma was frozen and stored at -80°C until used for analysis. A portion of each blood sample was used for blood glucose determination using an Abbott Freestyle Lite glucometer and glucose strips (Abbott Diabetes Care, Inc.; Alameda, CA). Where appropriate, determination of blood β-hydroxybutyrate levels was performed using an Abbott Precision Xtra meter and ketone strips (Abbott Diabetes Care, Inc.). At the end of each study, animals were fasted and bled, as described above, and then sacrificed. Inguinal and perigonadal fat pads, as well as liver, were harvested by surgical resection, weighed, and either prepared for histological analyses, as described below, or flash frozen and stored at -80°C.

**Liver Histological Analyses**

Histological analyses to assess liver steatosis were performed as previously described (Li *et al.*, 2003; Malloy *et al.*, 2013). Briefly, liver samples were fixed in 10% formalin overnight at 4^o^C. On the following day, samples were transferred to 70% ethanol and stored at 4^o^C prior to paraffin-embedding. Embedded samples were then sectioned (4–5 μm thick), stained with hematoxylin and eosin, and visualized microscopically. The resulting micrographs were examined blindly by a certified veterinary pathologist and steatosis grading was determined by averaging the grades of ten high-power (200x) magnification fields per sample. The grading scheme was as follows: 0 = no fatty hepatocytes; 1 = fatty hepatocytes occupying <33% of the hepatic parenchyma; 2 = fatty hepatocytes occupying 34%–66% of the parenchyma; 3 = fatty hepatocytes occupying >66% of the parenchyma. Six biological replicates were performed for each feeding condition.

**Metabolic Tolerance Tests**

Glucose tolerance tests (GTTs) and insulin tolerance tests (ITTs) were performed essentially as described (Ables *et al.*, 2012; Benede-Ubieto *et al.*, 2020). Prior to testing, mice were fasted for 4 hrs with free access to water. For GTTs, mice received intraperitoneal (IP) injections with 20% glucose (Sigma-Aldrich; St. Louis, MO) in 0.9% saline (Moltox, Inc.; Boone, NC) at a 1.5 g/kg dose. For ITTs, mice received IP injections of 0.25 U/ml of insulin (Eli Lilly & Co.; Indianapolis, IN) in 0.9% saline solution at a 0.5 U/kg dose. Mice were bled from a tail clip and blood glucose was measured immediately before injection and at 15, 30, 60, 90, and 120 min post-injection using an Abbott Freestyle Lite glucometer and glucose strips (Abbott Diabetes Care, Inc.). Six biological replicates were performed for each feeding condition.

**Plasma Amino Acid Analyses**

Measurements of the relative plasma levels of the sulfur-containing amino acids methionine, cysteine, and homocysteine were performed using fluorometric assay kits obtained commercially (Sigma-Aldrich). All tests were performed according to the manufacturer’s recommendations and measured using a Molecular Devices SpectraMax M5 Microplate Reader (Molecular Devices LLC; San Jose, CA). Two technical replicates were performed for each sample.

**Statistical Analyses**

Data were analyzed using the software package Prism 8 (GraphPad Software; La Jolla, CA). For analyses of longitudinal body condition and circulating analyte measurements, we performed ordinary two-way ANOVA. For analyses of terminal body condition and circulating analyte measurements (including plasma amino acids), we performed ordinary one-way ANOVA. For analyses of steatosis grade and metabolic tolerance tests, we performed unpaired two-tailed t-tests. *Post-hoc* testing of ANOVA results was performed using Fisher’s least significant difference tests and a family-wise significance level of 0.05 (95% confidence interval). Where appropriate, statistically significant differences are indicated, as follows: *, p<0.05; **, p<0.01; ***, p<0.001; ****, p<0.0001.

**Supplementary Table 1 - Composition of Mouse High-Fat Diets**

|  | **Diet 1 (CF; 0.86%)** | | **Diet 2 (MR; 0.12% MET)** | | **Diet 3 (0% MET)** | |
| --- | --- | --- | --- | --- | --- | --- |
|  | **g%** | ***kcal%*** | **g%** | ***kcal%*** | **g%** | ***kcal%*** |
| Protein | 15 | *12* | 15 | *12* | 15 | *12* |
| Carbohydrate | 41 | *31* | 41 | *31* | 41 | *31* |
| Fat | 34 | *57* | 34 | *57* | 34 | *57* |
| **Total** | **100** | ***100*** | **100** | ***100*** | **100** | ***100*** |
| **kcal/g** |  | ***5.3*** |  | ***5.3*** |  | ***5.3*** |
|  | | | | | | |
| **Ingredient** | **g** | ***kcal*** | **g** | ***kcal*** | **g** | ***kcal*** |
| L-Arginine | 11.2 | *45* | 11.2 | *45* | 11.2 | *45* |
| L-Histidine-HCl-H_2_O | 3.3 | *13* | 3.3 | *13* | 3.3 | *13* |
| L-Isoleucine | 8.2 | *33* | 8.2 | *33* | 8.2 | *33* |
| L-Leucine | 11.1 | *44* | 11.1 | *44* | 11.1 | *44* |
| L-Lysine | 14.4 | *58* | 14.4 | *58* | 14.4 | *58* |
| DL-Methionine | 8.86 | *35* | 1.24 | *5* | 0 | *0* |
| L-Phenylalanine | 11.6 | *46* | 11.6 | *46* | 11.6 | *46* |
| L-Threonine | 8.2 | *33* | 8.2 | *33* | 8.2 | *33* |
| L-Tryptophan | 1.8 | *7* | 1.8 | *7* | 1.8 | *7* |
| L-Valine | 8.2 | *33* | 8.2 | *33* | 8.2 | *33* |
| L-Glutamic Acid | 27.83 | *111* | 35.5 | *142* | 35.5 | *142* |
| L-Glutamine | 0 | *0* | 0 | *0* | 1.24 | *5* |
| Glycine | 23.3 | *93* | 23.3 | *93* | 23.3 | *93* |
|  | | | | | | |
| Corn Starch | 35 | *140* | 35 | *140* | 35 | *140* |
| Maltodextrin | 125 | *500* | 125 | *500* | 125 | *500* |
| Dextrose | 50 | *200* | 50 | *200* | 50 | *200* |
| Sucrose | 150 | *600* | 150 | *600* | 150 | *600* |
| Cellulose | 50 | *0* | 50 | *0* | 50 | *0* |
| Corn Oil | 46 | *414* | 46 | *414* | 46 | *414* |
| Lard | 257 | *2313* | 257 | *2313* | 257 | *2313* |
|  | | | | | | |
| Mineral Mix S10001 | 35 | *0* | 35 | *0* | 35 | *0* |
| Vitamin Mix V10001 | 10 | *40* | 10 | *40* | 10 | *40* |
| Choline Bitartrate | 2 | *0* | 2 | *0* | 2 | *0* |
| Dye | 0.05 | *0* | 0.05 | *0* | 0.05 | *0* |
| **Total** | **898** | ***4759*** | **898** | ***4759*** | **898** | ***4759*** |

| **Supplementary Table 2 - Key Resources** | | | | |
| --- | --- | --- | --- | --- |
| **Reagent type (species) or resource** | **Designation** | **Source or reference** | **Identifiers** | **Additional information** |
| Genetic reagent (*M. musculus*) | C57BL/6J | Jackson Laboratory | Stock No:000664 |  |
| Commercial assay or kit | IGF-1 Quantikine ELISA kit | R&D Systems | Cat. No:MG100 |  |
| Commercial assay or kit | Adiponectin Quantikine ELISA kit | R&D Systems | Cat. No:MRP300 |  |
| Commercial assay or kit | Leptin Quantikine ELISA kit | R&D Systems | Cat. No:MOB00B |  |
| Commercial assay or kit | FGF-21 ELISA kit | Millipore Corp. | Cat. No:EZRMFGF21-26K |  |
| Commercial assay or kit | Insulin ELISA kit | ALPCO Diagnostics | Cat. No:80-INSMS-E01 |  |
| Commercial assay or kit | Methionine Assay kit | Sigma-Aldrich | Cat. No:MAK347 |  |
| Commercial assay or kit | Cysteine Assay kit | Sigma-Aldrich | Cat. No:MAK255 |  |
| Commercial assay or kit | Homocysteine Assay kit | Sigma-Aldrich | Cat. No:MAK354 |  |
| Software, algorithm | GraphPad Prism | GraphPad Software | RRID:SCR_002798 | Version 8.0.0 |

**SUPPLEMENTARY FIGURES**

**Figure S1. Dietary Regimens**

A diagrammatic representation is shown for one week of control feeding (CF), continuous methionine restriction (MR), and two increasingly stringent variants of intermittent methionine restriction (IMR1 and IMR2). Additionally, a modified form of IMR2 is depicted (IMR2-A) that features alternate-day rather than contiguous-day dietary restriction. Percent values shown represent the concentration of methionine present in each diet, as well as the 7-day average (AVG) for each regimen. Black arrows indicate periods of methionine-replete feeding (0.86% methionine), gray arrows indicate periods of low methionine feeding (0.12% methionine), and white arrows indicate periods of methionine-free feeding (0% methionine).

**Figure S2. Expanded Food Consumption Measurements for Male Mice Subjected to IMR and Continuous MR**

Comparisons over time of average values for (A-B) food consumption, and (C-D) food consumption normalized to total body mass for control-fed (CF; black circles) or continuously methionine-restricted (MR; red squares) male mice, as well as animals subjected to IMR (IMR1; light blue triangles) or a more stringent IMR regimen (IMR2; dark blue triangles). For panels A and C, each value represents the average of two time-points per week (*e.g.*, Day 4 and Day 7) that correspond to the ends of the replete and restricted periods, respectively, for intermittently methionine-restricted mice. In contrast, panels B and D are expanded versions of these graphs that show values for all time-points. For all panels, bars denote standard error of the mean (SEM). N=4 for all groups.

**Figure S3. Fat Depot and Liver Sizes Normalized to Lean Body Mass for Male Mice Subjected to IMR and Continuous MR**

Normalized values at conclusion of the experiments are shown for (A) mass of inguinal fat pads, (B) mass of perigonadal fat pads, and (C) liver mass for male mice that were control-fed (CF) or continuously methionine-restricted (MR), as well as animals subjected to IMR (IMR1) or a more stringent IMR regimen (IMR2). Bars denote standard error of the mean (SEM). Statistically significant differences (as compared with the corresponding CF values) are indicated (*, p<0.05; **, p<0.01; ***, p<0.001; ****, p<0.0001). Statistically significant differences between MR and IMR2 values are either indicated (**, p<0.01) or absent (ns). N=4 for all groups.

**Figure S4. Expanded Food Consumption Measurements for Female Mice Subjected to IMR and Continuous MR**

Comparisons over time of average values for (A-B) food consumption, and (C-D) food consumption normalized to total body mass for control-fed (CF; black circles) or continuously methionine-restricted (MR; red squares) female mice, as well as animals subjected to stringent IMR (IMR2; blue triangles) or animals fed similarly to IMR2 animals but provided methionine in their drinking water (IMR2 + MET; gray triangles). For panels A and C, each value represents the average of two time-points per week (*e.g.*, Day 4 and Day 7) that correspond to the ends of the replete and restricted periods, respectively, for intermittently methionine-restricted mice. In contrast, panels B and D are expanded versions of these graphs that show values for all time-points. For all panels, bars denote SEM. N=8 for all groups.

**Figure S5. Fat Depot and Liver Sizes Normalized to Lean Body Mass for Female Mice Subjected to IMR and Continuous MR**

Normalized values at conclusion of the experiments are shown for (A) mass of inguinal fat pads, (B) mass of perigonadal fat pads, and (C) liver mass for female mice that were control-fed (CF), continuously methionine-restricted (MR), subjected to stringent IMR (IMR2), or fed similarly to IMR2 animals but provided methionine in their drinking water (IMR2 + MET). Bars denote SEM. Statistically significant differences (as compared with the corresponding CF values) are indicated (*, p<0.05; ***, p<0.001; ****, p<0.0001). No statistically significant differences between MR and IMR2 values were observed (ns). N=8 for all groups.

**Figure S6. Expanded Food Consumption Measurements for Female Mice Subjected to Contiguous-Day IMR and Alternate-Day IMR**

Comparisons over time of average values for (A-B) food consumption, and (C-D) food consumption normalized to total body mass for female mice that were control-fed (CF; black circles), subjected to stringent IMR (IMR2; blue triangles), or fed a modified IMR2 regimen featuring alternating days of methionine restriction and repletion (IMR2-A; green squares). For panels A and C, each value represents weekly average food consumption. In contrast, panels B and D are expanded versions of these graphs that show all food consumption measurements. For all panels, bars denote SEM. N=8 for all groups.

**Figure S7. Fat Depot Sizes Normalized to Lean Body Mass for Female Mice Subjected to Contiguous-Day IMR and Alternate-Day IMR**

Normalized values at conclusion of the experiments are shown for (A) mass of inguinal fat pads and (B) mass of perigonadal fat pads for female mice that were control-fed (CF), subjected to stringent IMR (IMR2), or fed a modified IMR2 regimen featuring alternating days of methionine restriction and repletion (IMR2-A). Bars denote SEM. Statistically significant differences (as compared with the corresponding CF values) are indicated (*, p<0.05; **, p<0.01). No statistically significant differences between IMR2 and IMR2-A values were observed. N=8 for all groups.

**REFERENCES**

Ables, G.P., Perrone, C.E., Orentreich, D., and Orentreich, N. (2012). Methionine-restricted C57BL/6J mice are resistant to diet-induced obesity and insulin resistance but have low bone density. PLoS One *7*, e51357.

Benede-Ubieto, R., Estevez-Vazquez, O., Ramadori, P., Cubero, F.J., and Nevzorova, Y.A. (2020). Guidelines and Considerations for Metabolic Tolerance Tests in Mice. Diabetes Metab Syndr Obes *13*, 439-450.

Li, Z., Yang, S., Lin, H., Huang, J., Watkins, P.A., Moser, A.B., Desimone, C., Song, X.Y., and Diehl, A.M. (2003). Probiotics and antibodies to TNF inhibit inflammatory activity and improve nonalcoholic fatty liver disease. Hepatology *37*, 343-350.

Malloy, V.L., Perrone, C.E., Mattocks, D.A., Ables, G.P., Caliendo, N.S., Orentreich, D.S., and Orentreich, N. (2013). Methionine restriction prevents the progression of hepatic steatosis in leptin-deficient obese mice. Metabolism *62*, 1651-1661.
